# Research protocol for delivering on the front line: a qualitative exploration of paramedics’ experiences of providing pre-hospital maternity care in the United Kingdom

**DOI:** 10.29045/14784726.2022.12.7.3.44

**Published:** 2022-12-01

**Authors:** Melissa Newman

**Affiliations:** University of Bath

**Keywords:** maternity, obstetrics, paramedics

## Abstract

**Background and aim:**

Maternity patients form a small but significant portion of paramedical workload and this research aims to pragmatically explore East of England Ambulance Service paramedics’ experiences of providing pre-hospital maternity care.

**Methods:**

Through semi-structured individual interviews, participants’ thoughts and feelings regarding obstetric confidence and competence will be explored. It will be investigated whether they think their training and equipment is adequate and what they would change about maternity patient management. Data will be thematically analysed and the findings used to improve paramedic training, with a view to improving their confidence and competence. In turn, this should improve patient experiences and outcomes.

## Background

### Ambulance service obstetric incidence

Childbirth is a normal physiological event; however, complications can arise. Its unpredictable nature can result in unplanned out-of-hospital births (UOOHBs), which are associated with neonatal hypothermia (Girsen et al., 2018), hypoglycaemia (Hadar et al., 2005) and jaundice (Di Benedetto et al., 1996), all of which increase the risk of intensive care admission. Additionally, there is a greater incidence of neonatal death (NND) in UOOHB (McLelland et al., 2018) compared with planned homebirths and hospital births. Mothers also suffer poorer outcomes, with a higher prevalence of perineal trauma and retained placenta, increasing risk of postpartum haemorrhage (PPH) (McLelland et al., 2014). Women birthing in paramedic care have up to a 1646% higher chance of PPH and 314% increased risk of shoulder dystocia compared to birthing in midwifery care (Ambulance Statistics, 2021; Royal Berkshire Hospital, 2020; Royal College of Obstetricians and Gynaecologists [RCOG], 2012). UK paramedics’ experiences of this are unresearched, hence this study will provide a novel contribution to knowledge.

In 2017, London Ambulance Service attended 2250 labouring women, delivered 800 babies and managed emergencies such as cord prolapse, breech birth, neonatal resuscitation (NNR), shoulder dystocia and PPH (London Ambulance Service, 2018). I submitted freedom of information requests (FOIRs) to every UK ambulance service to obtain obstetric incidence data for the years 2015–2021. Responses indicate that maternity patients form a small but significant portion of calls, with crews responding to approximately 60,000 obstetric calls annually.

APGAR (activity, pulse, grimace, appearance, respiration) scores assess how well babies tolerated birth and are coping extra-uterine (National Institute for Health and Care Excellence [NICE], 2020). Normal APGARs are > 7 and research demonstrates that low scores are inversely correlated with neonatal mortality (Moster et al., 2001), cerebral palsy and epilepsy (Persson et al., 2018). Of the babies in McLelland et al.’s (2018) study, 23% had APGARs ≤ 7 and while 1.4% of babies born in hospital or at planned homebirths score ≤ 7 at five minutes old (NHS Digital, 2020), no one-minute data exist, so UOOHB rates cannot be compared to planned homebirth/hospital rates. However, the finding that 23% of babies had APGARs ≤ 7 is a limitation of their study as scoring was inaccurate. APGARs should be recorded at one and five minutes old and the first APGAR was calculated whenever paramedics arrived on-scene if the baby was already born (59% of cases), and the second upon hospital arrival. As some on-scene times were two hours and transfer times three hours, babies could be five hours old when their five-minute APGAR was recorded. Therefore, rates of low APGAR scores at five minutes old may be higher than documented. The high incidence of low APGAR in this Australian study could explain the high NND rate of 2.7% versus 0.2% in hospital births / planned homebirths (Australian Institute of Health and Welfare, 2020). Some of the low APGAR and high NNR and NND rates could be explained by paramedics administering one third of women drugs which are contra-indicated in labour due to their severe neonatal respiratory depressive effect (McLelland et al., 2018).

Although 1.2% of babies in McLelland et al.’s (2018) Australian study required resuscitation, paramedics administered oxygen to 19.1% and suctioned 10.2% airways. This could be because anxious inexperienced paramedics suction faster and more unnecessarily compared to experienced paramedics (Vagle et al., 2019). Furthermore, although 6.2% of mothers in Flanagan et al.’s (2017) study suffered PPH, paramedics performed fundal massage on 31% who birthed their placentas, demonstrating inappropriate use of this technique on many mothers. Fundal massage was also practised on 19.7% of women who had not birthed their placentas, despite normal vital signs and no evidence of PPH. Similarly, McLelland et al. (2018) found that paramedics performed fundal massage on 23.5% of women despite only 6.5% requiring it. Fundal massage prior to placental delivery is contra-indicated as it can cause retained products and PPH (Jacob, 2018), however despite this, 20% of fundal massage was performed prior to placental delivery (McLelland et al., 2018). Discrepancies between evidence-based medicine and practice regarding APGAR, NNR and PPH indicate a knowledge gap, confusion over terminology or inaccurate record keeping.

As paramedics are not equipped with neonatal thermometers, newborn temperature is measured with tympanic thermometers, which are designed for adults as ear vernix causes inaccurate results. McClelland et al.’s (2019) service evaluation of the North East Ambulance Service found that of 168 newborns, paramedics measured 1% of blood sugars, 8% APGARS, 10% temperatures, 21% heart rates and 23% respiratory rates. No babies had all five observations recorded. Babies born in UOOHB settings are more susceptible to hypothermia, which increases their risk of requiring intensive care admission (Girsen et al., 2018), however if temperature is not measured, hypothermic babies cannot be identified. All babies whose temperatures were taken in Flanagan et al.’s (2017) study were hypothermic; however it is unknown whether this was due to inaccurate testing or the increased risk in UOOHB settings. It must be noted that only 2.4% of babies had their temperatures taken, highlighting how few babies’ temperatures are checked.

O’Hara et al. (2015) found that UK paramedics fear litigation and job loss so when not adequately equipped to assess patients, some purchase their own equipment, the maintenance and calibration of which is not monitored. These findings inform the need for research exploring whether paramedics possess the knowledge and medical tools to confidently manage maternity patients.

### Decision making

Paramedics make complex time-sensitive decisions in dynamic environments during emotional and uncertain circumstances and research indicates that poor obstetric emergency management can result in post-traumatic stress and depression for both paramedics and mothers (Flanagan et al., 2019; Vagle et al., 2019). Obstetric litigation continues to rise, with two claims a week settled for brain-injured infants (Department of Health [DoH], 2017). Between 2010 and 2020, 11% of clinical negligence cases in England were obstetric related, amassing to almost £518.5 million (NHS Resolution, 2021). I submitted FOIRs to each UK ambulance service, NHS Resolution and the Office for National Statistics but due to COVID-19 pandemic delays the dataset remains incomplete. However, one service responded that over 75% of litigation costs in 2019–2020 were obstetric-related, totalling £210,475. Obstetric litigation is expensive for the ambulance service as claim costs are disproportionately high in comparison to number of cases, for example one claim cost £3.5 million (Dobbie & Clarke, 2008). Over three-quarters of brain injuries and birth-related deaths are avoidable by appropriate care and treatment during birth (DoH, 2017). As UOOHB increases risk of PPH, 9% of maternal deaths are attributable to PPH (Knight et al., 2020) and the government strives to halve stillbirths, neonatal brain injuries, NNDs and maternal deaths by 2025 (DoH, 2017), it is important to explore paramedics’ experiences of providing pre-hospital maternity care to identify whether improvements can be made.

UK-wide inconsistency exists regarding the pre-arrival instructions emergency call handlers give maternity patients. For example, postcode dependent, women are advised to labour on their left side if the local ambulance service follows UK-based NHS Pathways algorithms, versus supine if they use America’s Advanced Medical Priority Dispatch System. The latter causes vena cava compression, reducing placental perfusion and increasing stillbirth risk, dyspnoea, dizziness and nausea (Stone et al., 2017). Further disparities include umbilical cord clamping, amniotic sac breaking and haemorrhage management, with some advice being non-evidence based. The Healthcare Safety Investigation Branch (HSIB) propound that although these algorithms are sometimes contributory to poor outcomes, it is impossible to quantify how many. Protocol deviation affects audit and accreditation (Lewis, 2021), hence paramedic decision making is influenced by which system their service uses. As change is challenging to incite, HSIB’s recommendations to remove algorithms advising mothers to lay on their backs and tie cords with shoelaces are yet to be implemented. They also recommend annual joint midwife and paramedic mandatory training regarding community obstetric emergency management and urge the importance of ambulance algorithms promoting best practice via consistent national guidance (Lewis, 2021).

McLelland et al. (2015) believed on-scene time was greater in women who birthed in paramedic presence because paramedics correctly identified imminent birth. However, this finding could also be interpreted as paramedics may misdiagnose the second stage of labour and therefore remain on-scene for longer while women labour then deliver, instead of transferring them to hospital. It is outside the paramedical scope of practice to internally examine women to ascertain cervical dilation, yet visually distinguishing between early, established and second stages of labour requires specialist skills that paramedics are not taught nor have adequate clinical exposure to due to the relative infrequency of obstetric callouts. Paramedics may not recognise warning signs or misinterpret others, for example assuming urge to push indicates imminent delivery so delaying conveyance to hospital despite this being an unreliable indicator as women with babies in occiput posterior positions experience urges to push in early labour (Borelli et al., 2013). Lack of skills and exposure can mean paramedics lack knowledge to base decisions on.

UK paramedics abide by Joint Royal Colleges Ambulance Liaison Committee (JRCALC) guidelines, which are not always evidence-based and sometimes contradict NICE and RCOG guidelines. For example, JRCALC (2021) encourages pushing and 30 seconds axial traction during shoulder dystocia management despite research showing both to be not only ineffective but dangerous as they further impact the shoulder on the pubic arch, possibly leading to brachial plexus injuries, Horner’s syndrome, neurological damage or death (Boyle, 2016). Hence, guidelines stress the importance of discouraging pushing and applying brief routine axial traction (RCOG, 2012). There are also discrepancies between JRCALC and NICE regarding placental delivery and placing premature babies in polythene bags. Lastly, JRCALC (2021) states that term babies should be placed skin-to-skin with mothers but babies < 37 weeks should be wrapped in a towel and placed next to mothers. This is despite skin-to-skin being most effective at minimising heat loss, of which premature babies are at greater risk (Safari et al., 2018). As paramedics rely on guidelines upon which to base decisions, and some of JRCALC’s recommendations are non-evidence based and detrimental, this could explain the high maternal and neonatal morbidity rates.

### Training

Training quantity and quality influences paramedic decision making. Obstetric emergencies are stressful even for multidisciplinary teams of experienced midwives and obstetricians. Personal investigation into the 46 UK universities that offer a paramedicine degree found that the average duration of obstetric training is three days, with some universities offering no teaching hours (University Responses, 2020). Universities with no contact hours expect students to undertake self-directed study, with no mechanisms for monitoring this. As many universities do not offer maternity placements, students may qualify without witnessing birth, obstetric emergencies or even practising on a model. In those that do offer maternity placements, the average length is 2.6 days and as medical and midwifery students have delivery suite priority, some paramedic students are placed in community or wards where they are unlikely to witness births or emergencies.

Of student paramedic education, 0.0–2.7% is devoted to obstetrics (University Responses, 2020) and oftentimes it is combined with gynaecological and paediatric education. As there are few similarities between children and pregnant women, this may suggest that care prioritises neonatal health rather than holistic maternal well-being. Some professors believe longer obstetric training should be mandatory (Evans, 2020), however the Health and Care Professions Council [HCPC] (2014), which accredits paramedic degrees, does not stipulate minimum standards of obstetric proficiency. It simply state paramedics must understand the key concepts of anatomy and physiology alteration throughout life, with no reference to obstetrics. Hence, each university creates their own curriculum, with training varying between institutions.

The HCPC (2014) requires paramedics to practise within the remit of their knowledge and skills, which, due to minimal training, could be limited. For example, paramedics in Flanagan et al.’s (2019) Australian study admitted to women their inexperience due to learning the basics from textbooks. Understandably, this concerned women. Research shows the responsibility and lack of skills and equipment leaves paramedics vulnerable and stressed when attending UOOHB. Many feel so inexperienced due to sometimes only a one-hour lecture; they perceive women to be at the mercy of nature and good outcomes attributable to luck rather than appropriate care (Vagle et al., 2019). Near-misses and fatalities sometimes prompt paramedics to seek their own training, as skills sessions are crucial in ensuring paramedics are up to date and competent in infrequently encountered situations (O’Hara et al., 2015; Vagle et al., 2019). Trust-organised training is sometimes cancelled due to service demand, meaning care is based on partial knowledge and as paramedics receive no feedback on their decision making, learning from experience is challenging.

### The problem

Some labouring women in Flanagan et al.’s (2019) study felt patronised, reprimanded, blamed and not believed by paramedics. At this frightening time they yearned for reassurance and empathy but were greeted with lack of consent and violation of privacy. They complained that the paramedical approach was too clinical as they maintained a distance rather than engaging. Due to these criticisms, some reported their trust in the ambulance service to be broken. It is crucial to consider service user perceptions of paramedic care, however only this study was yielded when literature searching and it is Australian, meaning these views cannot be generalised. Furthermore, it must be acknowledged that paramedics are generalists rather than specialists and the ambulance service’s aim is to safely manage emergencies and convey patients to hospital. Heavy criticism is unfair as paramedics cannot be expected to possess the knowledge and skills of a midwife.

Understanding paramedics’ and patients’ feelings infor-med this protocol’s research question, aims and objectives. A paucity of evidence exists surrounding UK paramedics’ experiences of providing pre-hospital maternity care and this study aims to address that.

### Research question

What are paramedics’ experiences of providing pre-hospital maternity care?

### Aim

To explore UK paramedics’ experiences of providing pre-hospital maternity care.

### Objectives

1. To explore paramedics’ attitudes to and beliefs about providing pre-hospital maternity care.
2. To identify challenges that paramedics face when responding to maternity calls.
3. To explore paramedics’ perceived confidence and competence in managing maternity patients.
4. To ascertain if paramedics feel their training equips them with sufficient knowledge to manage obstetric emergencies.
5. To investigate whether paramedics feel they carry the necessary equipment to enable them to effectively manage obstetric emergencies.

### Theoretical framework

The flexible nature of pragmatism is most suitable for this research as focus is on the research question and consequences of the study rather than the methodology. Pragmatists state reality to be constantly evolving rather than static, and this belief is important as this research aims to gain an insight into paramedics’ experiences of providing pre-hospital maternity care. The pragmatic paradigm believes the world to be changed by actions and an objective of this study is for positive changes to be implemented from the findings; thus, pragmatism is the most appropriate approach.

## Methods

### Inclusion criteria

- East of England Ambulance Service Trust (EEAST) paramedic contract.
- Minimum of three self-declared episodes of providing pre-hospital maternity care as a qualified paramedic, where ‘pregnancy/labour/childbirth/postnatal’ was the primary complaint.
- All ages and genders welcome.

### Recruitment

EEAST will advertise my study poster in the staff newsletter they email to paramedics. This will encourage them to contact me if they have queries or are interested in partaking. I will then email the participant information leaflet to those who contact me. Additionally, EEAST will advertise the study on Twitter. Data collection runs from 21 February 2022 to 31 July 2022. Once six and then 12 participants have been recruited, I will review their demographics to ensure balanced representation. Should a subgroup be underrepresented, for example female paramedics, EEAST research department will target via email underrepresented paramedics. Participants will be interviewed on a first come first served basis so once the target sample has been fulfilled, recruitment will cease.

### Sample size and technique

To prevent participation for personal benefit, no incentive will be provided. A total of 12–24 participants will be recruited via volunteer sampling. Much debate exists over what constitutes an adequate sample size in qualitative research and a recent systematic review found little or no sample size justification among health studies (Vasileiou et al., 2018). However, it is widely propounded that 12 participants should enable data saturation (Braun & Clarke, 2013; Guest et al., 2006). Considering this and in addition to observing the sample sizes of many high-quality studies, this study will recruit 12–24 participants via volunteer sampling.

### Data collection

The demographics to be collected are detailed in Table 1. Due to the COVID-19 pandemic, the safest method of facilitating the semi-structured interviews is virtually via Microsoft Teams. They will be recorded via Dictaphone. Each interview is anticipated to last 30–60 minutes and following each I will record field notes. Data will be stored as MP3 files. Participants will be made aware that they have two weeks post-interview to withdraw, after which time I will anonymise the data and thus withdrawal is not possible.

**Table 1.** Demographics to be collected.

**1**	Gender
**2**	Age
**3**	Agenda for Change band
**4**	Years qualified
**5**	Approximate quantity of maternity experiences where pregnancy/labour/birth/postnatal was the primary complaint: 3–8, 9–14, 15–20, 21–26, 27+
**6**	Method of qualification and additional post-graduate qualifications held
**7**	Post-qualification obstetric education undertaken

### Data analysis

Analysis will take place between 28 March 2022 and 4 September 2022. Via NVivo, data will be transcribed verbatim and be manually reviewed by myself for accuracy. As the pragmatic paradigm is flexible, manual thematic analysis using an inductive approach will be used to analyse the data. This will be conducted by me and overseen by my supervisors. Thematic analysis is an iterative process that requires the researcher to be familiar with the transcripts (Fugard & Potts, 2019) but as reflexive and objective as possible to minimise bias.

### Reflexivity

I am a midwife and Doctoral student who has undertaken a number of third manning shifts with the ambulance service in previous years. Practising alongside paramedics both during these shifts and as a community midwife, and discussing pre-hospital maternity care with paramedic peers is what inspired this research.

### Expected outcomes

The findings will be shared with EEAST staff via their e-newsletter. The study will be published in a journal and may also be shared at a conference. It is hoped that the research findings will be used to improve paramedic training, thus benefitting both paramedics and patients.

### Ethical considerations

This Doctoral study is self-funded and there are no conflicts of interest. The research was granted university ethical approval (reference: EP 20/21 099) in November 2021, Health Research Authority approval (IRAS project ID: 304635) in January 2022 and East of England Ambulance Service Research and Development approval. Informed consent will be obtained by providing participants with the participant information sheet and allowing them to ask questions before signing the consent form. Beforehand, the interview participants will be reminded of their right to withdraw at any point up to two weeks post-interview and I will reiterate this immediately after the interview as well. They do not need to provide a reason and will suffer no consequences.

The interview questions do not seek to cause psychological distress and the patient information sheet reassures participants they do not have to answer anything they do not want to. This will be reiterated immediately before the interview. While no question asks participants to discuss traumatic experiences, they may choose to. To ensure participants feel well supported should distress arise, I will email them the post-interview information leaflet as soon as the interview ends. This contains contact details of three independent support organisations and reminds paramedics that they can seek managerial or team leader support. As the interviews will be virtual, participants may have family or friend support with them, however they also may be alone. If this is the case and a participant appears distressed, I will encourage them to contact a trusted colleague, friend or family member for support. I will also ask for consent to alert the participant’s manager so they can check in on their well-being. The participant information leaflet and consent form make it clear that participants should not disclose criminal activity or misconduct as this may necessitate confidentiality being broken in order to report it to the relevant legal authorities. I will remind participants of this immediately before the interview.

Transcripts will be ‘cleaned’ by myself to remove identifiable details of both participants and patients. Identifiable personal data and anonymised data will be stored in a password-protected encrypted folder on the University of Bath X:Drive and access will be restricted to my academic supervisor and me. Consent forms will be digitised, personal data only kept for as long as necessary for the project and identifiable data stored in a separate encrypted folder from study data. To maintain anonymity, audio recordings and raw transcripts will be destroyed following publication but to facilitate reproducibility and be of possible value to future research, anonymised transcripts and signed consent forms will be uploaded to the University of Bath Research Data Archive and preserved for 10 years following publication.

## Author contributions

MN acts as the guarantor for this article.

## Conflict of interest

None declared.

## Funding

None.

## References

[bibr_1] Ambulance Statistics. (2021). *Freedom of information responses*. Unpublished.

[bibr_2] Australian Institute of Health and Welfare. (2020). *Stillbirths and neonatal deaths in Australia*. AIHW. https://www.aihw.gov.au/reports/mothers-babies/stillbirths-and-neonatal-deaths-in-australia/contents/overview-of-perinatal-deaths.

[bibr_3] Borelli S. E. Locatelli A. & Nespoli A. (2013). Early pushing urge in labour and midwifery practice: A prospective observational study at an Italian maternity hospital. *Midwifery*, 8, 871–875. 10.1016/j.midw.2012.09.010 23415319

[bibr_4] Boyle M. (2016). *Emergencies around childbirth* (3rd ed.). Routledge.

[bibr_5] Braun V. & Clarke V. (2013). *Successful qualitative research*. SAGE Publications.

[bibr_6] *Data Protection Act 2018*, c. 12. The Stationery Office.

[bibr_7] Department of Health. (2017). *Safer maternity care*. DoH.

[bibr_8] Di Benedetto M. R. Piazze J. J. Unfer V. Ouatu D. Pollastrini L. Vozzi G. Marzano P. F. & Anceschi M. M. (1996). An obstetric and neonatal study on unplanned deliveries before arrival at hospital. *Clinical and Experimental Obstetrics and Gynaecology*, 23(2), 108–111. 8737624

[bibr_9] Dobbie A. & Clarke M. (2008). A descriptive review and discussion of litigation claims against ambulance services. *Emergency Medical Journal*, 25(7), 455–458. 10.1136/emj.2007.052324 18573971

[bibr_10] Evans B. (2020). Paramedic obstetric curriculum. University of Sunderland. Unpublished.

[bibr_11] Flanagan B. Lord B. & Barnes M. (2017). Is unplanned out-of-hospital birth managed by paramedics ‘infrequent’, ‘normal’ and ‘uncomplicated’? *BMC Pregnancy and Childbirth*, 17(436), 183–191. 29273024 10.1186/s12884-017-1617-9 PMC5741876

[bibr_12] Flanagan B. Lord B. Reed R. & Crimmins G. (2019). Women’s experience of unplanned out-of-hospital birth in paramedic care. *BMC Pregnancy and Childbirth*, 19(54), 234–241. 31856736 10.1186/s12884-019-2613-z PMC6923941

[bibr_13] Fugard A. & Potts H. W. W. (2019). *Thematic analysis*. SAGE Publications.

[bibr_14] Girsen A. I. Mayo J. A. Lyell D. J. Blumenfeld Y. J. Stevenson D. K. El-Sayed Y. Y. Shaw G. M. & Druzin M. L. (2018). Out-of-hospital births in California 1991–2011. *Journal of Perinatology*, 38, 41–45. 29120453 10.1038/jp.2017.156

[bibr_15] Guest G. Bunce A. & Johnson L. (2006). How many interviews are enough? An experiment with data saturation and variability. *Field Methods*, 18(1), 59–82.

[bibr_16] Hadar A. Rabinovich A. Sheiner E. Landau D. Hallak M. & Mazor M. (2005). Obstetric characteristics and neonatal outcome of unplanned out-of-hospital term deliveries: A prospective, case-control study. *The Journal of Reproductive Medicine*, 50(11), 832–836. 16419631

[bibr_17] Health and Care Professions Council. (2014). *The standards of proficiency for paramedics*. HCPC. https://www.hcpc-uk.org/standards/standards-of-proficiency/paramedics/.

[bibr_18] Jacob A. (2018). *A comprehensive textbook of midwifery and gynecological nursing* (5th ed.). Jaypee Brothers Medical Publishers.

[bibr_19] Joint Royal Colleges Ambulance Liaison Committee. (2021). *JRCALC clinical guidelines*. Class Professional Publishing.

[bibr_20] Knight M. Bunch K. Tuffnell D. Shakespeare J. Kotnis R. Kenyon S. Kurinczuk J. J. (Eds.) on behalf of MBRRACE-UK. (2020). *Saving lives, improving mothers’ care: Lessons learned to inform maternity care from the UK and Ireland confidential enquiries into maternal deaths and morbidity 2016–18*. National Perinatal Epidemiology Unit, University of Oxford.

[bibr_21] Lewis S. (2021). *Introduction to HSIB investigation*. Healthcare Safety Investigation Branch. https://hsib-kqcco125-media.s3.amazonaws.com/assets/documents/Slide_Deck_Master_-_Ambulance_Webinar_21_June_2021.pdf.

[bibr_22] London Ambulance Service. (2018). *A world class ambulance service for a world class city: Strategy 2018/19–2022/23*. LAS. https://www.londonambulance.nhs.uk/wp-content/uploads/2018/07/London-Ambulance-Service-Strategy-2018-2023.pdf.

[bibr_23] McClelland G. Burrow E. & McAdam H. (2019). Babies born in the pre-hospital setting attended by ambulance clinicians in the north east of England. *British Paramedic Journal*, 4(3), 43–48. 33447150 10.29045/14784726.2019.12.4.3.43 PMC7783920

[bibr_24] McLelland G. McKenna L. Morgans A. & Smith K. (2018). Epidemiology of unplanned out-of-hospital births attended by paramedics. *BMC Pregnancy and Childbirth*, 18(15), 24–31. 29310618 10.1186/s12884-017-1638-4 PMC5759287

[bibr_25] McLelland G. Morgans A. & McKenna L. (2015). Victorian paramedics’ encounters and management of women in labour: An epidemiological study. *BMC Pregnancy and Childbirth*, 15(13), 113–120. 25652103 10.1186/s12884-015-0430-6 PMC4332426

[bibr_26] McLelland G. E. Morgans A. E. & McKenna L. G. (2014). Involvement of emergency medical services at unplanned births before arrival to hospital: A structured review. *Emergency Medical Journal*, 31(4), 345–350. 10.1136/emermed-2012-202309 23417265

[bibr_27] Moster D. Lie R. Irgens L. Bjerkedal T. & Markestad T. (2001). The association of APGAR score with subsequent death and cerebral palsy: A population-based study in term infants. *Journal of Pediatrics*, 138(6), 798–803. 11391319 10.1067/mpd.2001.114694

[bibr_28] National Institute for Health and Care Excellence. (2020). *Intrapartum care*. NICE. https://pathways.nice.org.uk/pathways/intrapartum-care.

[bibr_29] NHS Digital. (2020). *NHS maternity statistics, England 2019–20*. NHS Digital. https://digital.nhs.uk/data-and-information/publications/statistical/nhs-maternity-statistics/2019-20/births.

[bibr_30] NHS Resolution. (2021). *Obstetric claims*. NHS Resolution. https://resolution.nhs.uk/wp-content/uploads/2021/07/FOI_5096_Obstetric-Claims.pdf.

[bibr_31] O’Hara R. Johnson M. Siriwardena N. Weyman A. Turner J. Shaw D. Mortimer P. Newman C. Hirst E. Storey M. Mason S. Quinn T. & Shewan J. (2015). A qualitative study of systemic influences on paramedic decision making: Care transitions and patient safety. *Journal of Health Services Research and Policy*, 20(1), 45–53. 10.1177/1355819614558472 25472989

[bibr_32] Persson M. Razaz N. Tedroff K. Joseph K. & Cnattingius S. (2018). Five and 10 minute APGAR scores and risks of cerebral palsy and epilepsy: Population based cohort study in Sweden. *BMJ*, 360, k207. https://doi.org/10.1136/bmj.k207. 29437691 10.1136/bmj.k207 PMC5802319

[bibr_33] Royal Berkshire Hospital. (2020). *Previous minor post-partum haemorrhage (PPH)*. https://www.royalberkshire.nhs.uk/media/gjsnou5p/previous-minor-post-partum-haemorrhage_dec20.pdf.

[bibr_34] Royal College of Obstetricians and Gynaecologists. (2012). *Shoulder dystocia (green-top guideline no. 42)*. RCOG. https://www.rcog.org.uk/globalassets/documents/guidelines/gtg_42.pdf. 10.1111/1471-0528.70258 42660544

[bibr_35] Safari K. Saeed A. A. Hasan S. S. & Moghaddam-Banaem, L. (2018). The effect of mother and newborn early skin-to-skin contact on initiation of breastfeeding, newborn temperature and duration of third stage of labor. *International Breastfeeding Journal*, 13, 32. https://doi.org/10.1186/s13006-018-0174-9. 30026787 10.1186/s13006-018-0174-9 PMC6048813

[bibr_36] Stone P. R. Burgess W. McIntyre J. P. R. Gunn A. J. Lear C. L. Bennet L. Mitchell E. A. & Thompson J. M. D. (2017). Effect of maternal position on fetal behavioural state and heart rate variability in healthy late gestation pregnancy. *The Journal of Physiology*, 595(4), 1213–1221. 27871127 10.1113/JP273201 PMC5309385

[bibr_37] University Responses. (2020). *Paramedic obstetric curriculum*. Unpublished.

[bibr_38] Vagle H. Haukeland G. T. Dahl B. Aasheim V. & Vik E. S. (2019). Emergency medical technicians’ experiences with unplanned births outside institutions: A qualitative interview study. *Nursing Open*, 6, 1542–1550. 31660182 10.1002/nop2.354 PMC6805291

[bibr_39] Vasileiou K. Barnett J. Thorpe S. & Young T. (2018). Characterising and justifying sample size sufficiency in interview-based studies: Systematic analysis of qualitative health research over a 15-year period. *BMC Medical Research Methodology*, 18(1), 148). https://doi.org/10.1186/s12874-018-0594-7. 30463515 10.1186/s12874-018-0594-7 PMC6249736

